# Incretin-Based Therapies Through the Decades: Molecular Innovations and Clinical Impact

**DOI:** 10.3390/medsci13040269

**Published:** 2025-11-14

**Authors:** Arthur Anatolievich Lee, Victoria Alexandrovna Khotina, Dmitry Alexandrovich Kashirskikh, Olga Evgenevna Voronko, Vagif Ali oglu Gasanov, Andrey Valentinovich Vasiliev

**Affiliations:** Koltzov Institute of Developmental Biology of Russian Academy of Sciences, Moscow 119334, Russia; d.a.kashirskikh@idbras.ru (D.A.K.); o.e.voronko@idbras.ru (O.E.V.); v.a.gasanov@idbras.ru (V.A.o.G.); a.v.vasilev@idbras.ru (A.V.V.)

**Keywords:** incretins, GLP-1, GIP, GLP-1RA, T2DM, obesity, cardiometabolic diseases

## Abstract

The study of incretins spans more than a century and has revealed their essential role in glucose homeostasis and metabolic regulation. This understanding has led to the development of incretin receptor agonists as highly effective pharmacological agents for the treatment of such cardiometabolic diseases as type 2 diabetes and obesity, showing substantial benefits in glycemic control, body weight reduction, and cardiometabolic outcomes. However, their use is limited by adverse events, most commonly gastrointestinal intolerance, along with ongoing safety concerns regarding pancreatic, renal, and ophthalmologic effects. Although incretin-based therapies have fundamentally reshaped the management of diabetes and obesity, continued innovation in drug design and delivery holds promise for expanding their applicability, improving patient adherence, and reinforcing their role as a cornerstone of metabolic disease management and beyond. This review summarizes the historical development, molecular design, and clinical relevance of incretin-based therapies, with particular emphasis on approved agents used in current clinical practice.

## 1. Introduction

Incretins are gut-derived peptide hormones that are secreted in response to nutrient intake and play a pivotal role in glucose homeostasis. The two principal incretins, glucagon-like peptide-1 (GLP-1) and glucose-dependent insulinotropic peptide (GIP), enhance insulin secretion from pancreatic β-cells in a glucose-dependent manner. Beyond their classic pancreatic effects, incretins exert multiple systemic actions that influence appetite regulation, gastric motility, cardiovascular function, renal physiology, and even neuroprotection.

Agonists of GLP-1 and GIP receptors have rightfully attracted significant scientific and clinical attention and have already secured their place in the treatment of severe cardiometabolic diseases, such as type 2 diabetes mellitus (T2DM) and obesity [1]. Cardiometabolic diseases also include cardiovascular diseases (CVDs) such as coronary artery disease and arterial hypertension, as well as dyslipidemia, insulin resistance, liver and kidney damage, which often accompany T2DM and reflect the systemic nature of metabolic disorders. These diseases remain a leading cause of morbidity and mortality worldwide. According to the International Diabetes Federation (IDF) Diabetes Atlas (2025), approximately 600 million adults aged 20–79 are currently living with diabetes. Projections indicate a dramatic rise, with this number expected to reach 853 million by 2045 [2]. Cardiovascular mortality is also expected to rise sharply by 73% between 2025 and 2050, reaching approximately 35.6 million deaths per year, with coronary artery disease remaining the leading cause [3].

The invention of GLP-1 receptor agonists (GLP-1RAs) was a major milestone in the treatment of T2DM. These pharmaceutical drugs provide significant glycemic control by stimulating insulin secretion, suppressing glucagon secretion. It also reduces body weight. Over time, the understanding of the role of incretins has expanded significantly: their systemic effects on the central nervous system (CNS), cardiovascular function, cognitive processes, and immune response have been identified. GLP-1 agonists and combination drugs demonstrate not only glucose-lowering and anorexigenic properties but also nephro- and cardioprotective effects, including blood pressure reduction, improved lipid profile, and reduced albuminuria, effects that are partially independent of glycemic reduction. Furthermore, accumulating evidence supports their neuroprotective potential, as GLP-1RAs have been shown to improve cerebral glucose hypometabolism, reduce neuroinflammation, and attenuate neuronal death in models of neurodegenerative diseases, suggesting possible therapeutic relevance beyond metabolic disorders.

Despite impressive progress, the widespread implementation of incretin therapy is limited by the high cost of treatment, limited availability in some regions, the need for injectable administration, and gastrointestinal side effects that impact treatment adherence. These barriers underscore the need for further drug optimization, including the development of more affordable oral formulations, improved delivery systems, and multi-agonists with an enhanced safety profile.

In the context of the global epidemic of obesity and metabolic diseases, incretin-based therapy has become a cornerstone of modern treatment. However, its potential can only be fully realized when integrated with lifestyle modifications, social and digital patient support programs, and an interdisciplinary approach aimed at achieving long-term clinical outcomes. This review is dedicated to the historical development, molecular design, and clinical significance of incretin-based drugs, with special attention to approved agents used in current practice and prospects for their further improvement. It also focuses on clinical studies in the context of cardiometabolic health.

## 2. The Evolution of the Incretin Concept: From Discovery to Modern Physiological Understanding

### 2.1. Early Premises

**Figure 1:**
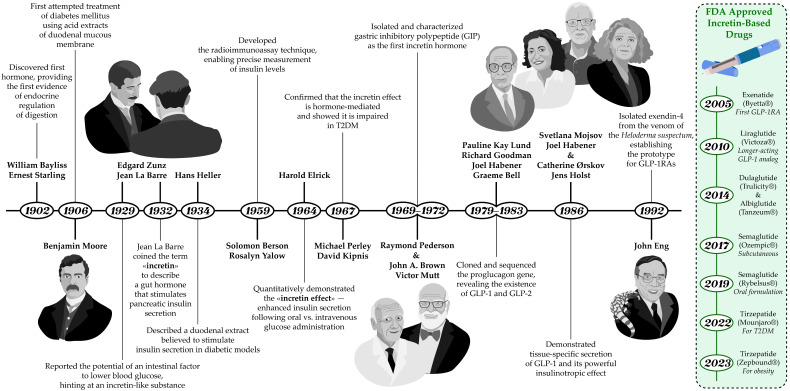
Timeline of key discoveries in incretin biology and therapy development. The figure highlights the identification of GIP and GLP-1 as incretin hormones, the demonstration of their insulinotropic effects, and the subsequent development of incretin-based drugs, including GLP-1RAs and DPP-4 inhibitors, which transformed diabetes treatment. The illustration of J. La Barre is shown from behind because his photograph is not available in the public domain on the internet, as is the case with the photograph of R. Pederson, who was decided not to be depicted.

The history of the discovery of incretins reflects the evolution of scientific understanding of the neuroendocrine regulation of digestion and carbohydrate metabolism (Figure 1). The complex relationship between the intestine and pancreatic function had long attracted the interest of researchers. The prevailing theory, championed by I. Pavlov, was that pancreatic secretion was regulated exclusively by the nervous system through vagal reflexes. However, an experiment in 1902 provided the first evidence for endocrine regulation of digestion, when W. Bayliss and E. Starling managed to demonstrate that a substance secreted by the intestinal mucosa, upon entering the bloodstream, stimulates pancreatic secretion [4]. In 1906 B. Moore was the first to successfully use a duodenal mucosal extract in the treatment of diabetes mellitus [5]. And in 1929, Belgian researchers J. La Barre and E. Zunz proposed the existence of an intestinal factor that could enhance pancreatic insulin secretion [6].

And in 1932, La Barre first coined the term “incretin” (from INtestine seCRETion INsulin) to describe an intestinal hormone that stimulates the endocrine function of the pancreas, including insulin release. Although a duodenal extract presumed to stimulate insulin secretion and lower blood glucose levels was also described by H. Heller in 1934, the incretin hypothesis remained controversial for many years due to the lack of reliable methods for accurately measuring insulin levels [7]. The situation changed in the late 1950s with the invention of radioimmunoassay by S. Berson and Rosalyn Yalow—a method that enabled accurate measurement of plasma insulin levels [8]. This technological breakthrough allowed researchers to explore incretins and their physiological effects in greater depth. With this new tool H. Elrick and colleagues conducted a 1964 study that provided the first quantitative evidence for gut-mediated regulation of glycemia, paving the way for the identification of incretin hormones [9]. Using plasma insulin assays, they directly demonstrated enhanced insulin secretion after oral glucose administration compared with intravenous glucose—despite identical glucose concentrations—an observation now known as the “incretin effect” [10]. In subsequent work published in 1967, M. Perley and D. Kipnis showed that this effect was mediated by endocrine factors of gastrointestinal origin. They found that obese individuals, both with and without diabetes, exhibited increased insulin secretion (hypersecretion), whereas in diabetic patients, regardless of weight, insulin secretion was significantly impaired [11].

### 2.2. Discovery of GIP

The search for intestinal hormones responsible for the incretin effect continued for decades and concluded in 1969 when a peptide was successfully isolated in the laboratory of professor V. Mutt. It was later named “gastric inhibitory polypeptide” or “GIP” in 1972 in studies conducted by R. Pederson and J. Brown [12]. Animal experiments confirmed the role of GIP as an incretin hormone—immunoneutralization with specific antibodies suppressed glucose-dependent insulin secretion [13]. Human studies also demonstrated its strong insulinotropic activity. In healthy volunteers, intravenous infusion of physiological doses of GIP together with glucose led to a greater rise in plasma immunoreactive insulin and improved glucose tolerance [14]. Studies in the 1980s using GIP immunoneutralization in rats and small-intestinal resection in humans did not completely abolish the incretin effect, indicating that another incretin hormone must exist [15,16].

### 2.3. Physiology and Functions of GIP

After its discovery, it was established that the primary physiological role of GIP is to stimulate insulin secretion in response to the intake of glucose and fats in the duodenum. Accordingly, the peptide was renamed “glucose-dependent insulinotropic polypeptide” while retaining the abbreviation GIP [17]. Although the molecular mechanisms of incretin signaling have been comprehensively described, a concise overview of the physiological actions of GIP is outlined below to provide context for its therapeutic relevance [18,19].

**Figure 2:**
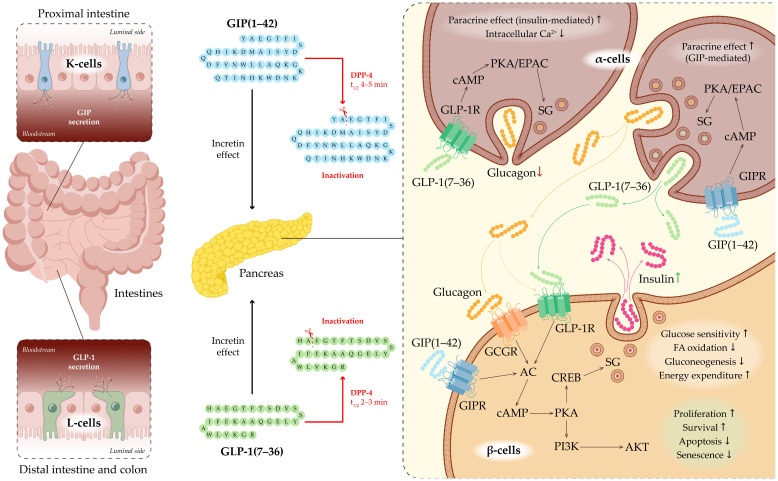
Incretin system and GLP-1 signaling in pancreatic islets. Schematic representation of the gastrointestinal tract highlighting enteroendocrine L-cells (green) that secrete GLP-1 and K-cells (light blue) that secrete GIP in response to nutrient intake. Secreted incretins act on the pancreas to regulate insulin and glucagon secretion. DPP-4 rapidly inactivates GLP-1 and GIP, limiting their biological activity. Binding of GLP-1 to its receptor GLP-1R activates intracellular cascades, leading to enhanced insulin secretion by β-cells and suppression of glucagon release by α-cells. Upon binding to GIPR the surface of β-cells, GIP directly augments insulin secretion in a glucose-dependent manner, potentiating insulin release only when plasma glucose concentrations are elevated. GIP indirectly enhances insulin secretion by stimulating pancreatic α-cell activity and promoting paracrine interactions between α- and β-cells. Abbreviations: ACh—Adenylate Cyclase; AKT—Protein Kinase B (PKB); cAMP—Cyclic Adenosine Monophosphate; CREB—cAMP Response Element-Binding Protein; DPP-4—Dipeptidyl Peptidase-4; EPAC—Exchange Protein Activated by cAMP; GCGR—Glucagon Receptor; GIP—Glucose-Dependent Insulinotropic Polypeptide; GIPR—GIP Receptor; GLP-1—Glucagon-Like Peptide-1; GLP-1R—GLP-1 Receptor; PI3K—Phosphoinositide 3-Kinase; SG—Secretory Granule.

GIP is secreted by K-cells located in the proximal small intestine, predominantly in the duodenum and jejunum (Figure 2). GIP secretion peaks within 30–60 min after glucose administration or ingestion of mixed meals [20]. The biologically active form, GIP(1–42), is generated from its 153-amino-acid precursor, a proprotein encoded by the GIP gene, through proteolytic processing [21]. GIP acts through the GIP receptor (GIPR), which belongs to the GPCR (G-protein-coupled receptor) family. GIPR is expressed in pancreatic β-cells, adipose tissue, and the CNS [22,23]. Upon binding to GIPR on the surface of β-cells, GIP activates the Gαs subunit, leading to stimulation of adenylate cyclase (AC) and a subsequent increase in intracellular cyclic AMP (cAMP) levels. Elevated cAMP activates protein kinase A (PKA) and exchange protein directly activated by cAMP (EPAC), thereby enhancing calcium influx through voltage-gated calcium channels (VGCCs) and priming insulin granules for calcium-dependent exocytosis. Through these mechanisms, GIP directly augments insulin secretion in a glucose-dependent manner, potentiating insulin release only when plasma glucose concentrations are elevated [18,19].

In addition to its direct effects on β-cells, GIP indirectly enhances insulin secretion by stimulating pancreatic α-cell activity and promoting paracrine interactions between α- and β-cells. Notably, α-cells are capable of producing not only glucagon but also GLP-1 via alternative post-translational processing of the proglucagon precursor. The co-release of glucagon and GLP-1 from α-cells is thought to further potentiate glucose-stimulated insulin secretion through GLP-1 receptor (GLP-1R)—and, to a lesser extent, glucagon receptor (GCGR)—mediated increases in cAMP signaling [19].

Beyond its pancreatic actions, GIP modulates hypothalamic activity to suppress appetite, regulates lipid metabolism by promoting triglyceride storage in adipocytes, and influences bone metabolism by enhancing bone formation [24,25,26]. GIP is rapidly inactivated—within approximately 5 min—by dipeptidyl peptidase-4 (DPP-4) cleavage to form the inactive GIP(3–42) fragment [20,27].

### 2.4. Discovery of GLP-1

In 1979, Pauline Kay Lund and R. Goodman discovered that anglerfish islet proglucagon messenger RNAs contained coding sequences for two glucagon-related peptides arranged in one tandem. The results of their work, published in 1982, revealed that the glucagon precursor gene encodes not one, but three biologically active peptides: glucagon itself and two previously unknown intestinal hormones [28]. In 1983, G. Bell sequenced the proglucagon gene from a hamster islet library. It was found that this gene encodes not only glucagon itself, which consists of 29 amino acids, but also other smaller peptides, including glicentin, oxyntomodulin, intermediate peptides, and two additional sequences with 50% homology to glucagon. These findings prompted the search for proglucagon processing products: glucagon-like peptide-1 and -2 (GLP-1 and GLP-2).

In 1987 Svetlana Moisov working in J. Habener’s laboratory demonstrated that synthetic N-terminally truncated GLP-1(7–37) is a more potent stimulator of insulin secretion, whereas GLP-1(1–37) has no effect on insulin secretion even at high concentrations [29]. Concurrently, Catherine Ørskov and J. Holst independently investigated GLP-1 and GLP-2 in the pancreas and small intestine of pigs [30]. They found that GLP-1 and GLP-2 are processed differently in various tissues and secreted in response to distinct stimuli. This discovery was crucial for understanding their roles in metabolism and digestion regulation. Subsequent research revealed that GLP-1 exhibits significant insulinotropic activity at physiological levels, establishing it as the second incretin known to science. Supporting this, J. Holst and colleagues demonstrated that synthetic analogs of this peptide also possess high insulinotropic activity [31]. As for GLP-2, it was found to lack insulinotropic effects but holds significant interest as an intestinal growth factor [32,33].

### 2.5. Physiology and Functions of GLP-1

Currently, it is known that GLP-1 is a peptide hormone generated through enzymatic cleavage of proglucagon, producing variants such as GLP-1(1-37), GLP-1(7-36) amide, and GLP-1(7-37). This hormone is synthesized by enteroendocrine L-cells located in the distal jejunum, ileum, and colon, as well as by α-cells in the pancreatic islets and neurons in the nucleus tractus solitarius of the medulla oblongata [34] (Figure 2). Its secretion is triggered by food intake and neuroendocrine stimuli, including activation of the vagus nerve [35,36]. GLP-1 enhances insulin secretion by pancreatic β-cells, suppresses glucagon production by α-cells, slows gastric emptying, and induces satiety via activation of central neuroendocrine pathways [37,38,39].

Through GIP and GLP-1 together, the incretin system is a major mechanism responsible for glucose-dependent stimulation of insulin secretion in response to nutrient intake. Following a meal, incretins account for up to 70% of postprandial insulin secretion [40]. Postprandial GLP-1 secretion occurs in two phases. The early phase begins within 10–15 min after food intake. Although the precise mechanisms underlying this rapid response remain under debate, it is thought to involve not only direct contact of nutrients with enteroendocrine cells but also additional factors such as neurotransmitters, other gut hormones, and parasympathetic innervation. The late phase starts after 10–15 min and may last up to 60 min, reflecting prolonged stimulation of intestinal neuroendocrine cells by nutrients. An inverse relationship between gastric emptying rate and GLP-1 secretion has been observed: higher GLP-1 levels are associated with slower gastric emptying, potentially serving as a feedback mechanism regulating its own secretion [41,42]. The biological action of GLP-1 is short-lived, with a plasma half-life of only 1–2 min under physiological conditions. Rapid inactivation occurs via the enzyme DPP-4, also known as CD26 [43].

The GLP-1R also belongs to the GPCR family and is expressed in multiple tissues and organs. Beyond the pancreas, it is also found in the cardiovascular system, gastrointestinal tract, CNS, and kidneys, indicating its role in regulating not only glucose metabolism but also other physiological processes. GLP-1R activation engages intracellular signaling cascades via Gs proteins, AC, PKA, and EPAC, leading to increased insulin secretion and maintenance of β-cell function: it enhances β-cell proliferation and survival, reduces apoptosis and senescence, improves glucose sensitivity, decreases fatty acid oxidation and gluconeogenesis, and promotes energy expenditure (Figure 2) [44,45,46]. Activation of PKA triggers the phosphorylation of cAMP response element-binding protein (CREB), which plays a role in activating the release of insulin-containing secretory granules [47]. GLP-1 also activates the phosphoinositide 3-kinase (PI3K)/protein kinase B (Akt) pathway, which is crucial for maintaining the survival and function of pancreatic β-cells [46]. In α-cells, GLP-1 signaling results in enhanced paracrine effects mediated by insulin, decreased intracellular calcium levels, and suppression of glucagon secretion [46].

Collectively, these effects contribute to improved pancreatic function, glycemic control, and metabolic homeostasis. Recognition of these multifaceted physiological effects spurred intensive research into therapeutic agents mimicking or enhancing incretin activity. The development of GLP-1RAs has since revolutionized the management of T2DM and related cardiometabolic disorders.

## 3. From Concept to Clinic: The Development of Incretin-Based Drugs

### 3.1. Main Strategies for Peptide Modification and Synthesis

Peptides—polymers of 2–50 amino acids—occupy a unique niche in pharmaceutical development. They are particularly promising for targeting complex biological systems, including intracellular protein–protein interactions, yet face significant pharmacokinetic limitations: poor membrane permeability, rapid proteolytic degradation, short half-life, and the need for parenteral administration [48].

Chemical peptide synthesis provides precise sequence control and enables site-specific structural modifications, making it the preferred method for producing peptides up to ~50 amino acids. This approach allows the incorporation of non-standard amino acids, such as D-isomers and N-methylated residues, that enhance proteolytic resistance and membrane permeability [49,50]. Common modifications include covalent backbone changes (e.g., N-terminal acetylation, C-terminal amidation), cyclization via disulfide bonds or lactam bridges, and conjugation with lipophilic groups to improve stability, bioavailability, and target specificity [51,52,53]. Attachment of polymers (e.g., polyethylene glycol (PEG)), lipids, or carbohydrates extends half-life by reducing renal clearance and enhancing solubility [54]. More advanced strategies, like peptoids and retro-inverso peptides, confer enzymatic stability under physiological conditions [55,56].

The most widely used method is solid-phase peptide synthesis employing Fmoc/tBu chemistry, which ensures high purity, reproducibility, and compliance with good manufacturing practice requirements [57]. For more complex or elongated molecules, native chemical ligation is employed, allowing the assembly of two or more peptide fragments while preserving biological activity [58].

### 3.2. Clinically Approved Drugs

Although metformin remains the preferred first-line therapy for T2DM according to the American Diabetes Association (ADA), incretin-based therapies have already reshaped the therapeutic landscape. In particular, initiation of a GLP-1RAs should be considered in patients with contraindications or intolerance to metformin, in those whose HbA1c exceeds the glycemic target by more than 1.5%, or in patients who fail to achieve target HbA1c within three months—especially in the presence of comorbid atherosclerotic CVD, chronic kidney disease, or heart failure [59].

Incretin analogs function as GLP-1RAs by activating the receptor and mimicking the action of endogenous GLP-1. Structurally, these analogs are GLP-1 mimetics, resembling native GLP-1 in amino acid sequence and three-dimensional conformation. The therapeutic application of native incretins is limited by rapid enzymatic degradation and a very short half-life, preventing sustained circulating levels. Consequently, pharmacological development has focused on extending the duration of action and stabilizing effects by optimizing pharmacokinetic profiles [60,61].

Modern GLP-1RAs can be categorized into two broad groups based on molecular origin. The first group comprises exendin-4–based agents, including exenatide and lixisenatide [60,62]. The second group consists of human GLP-1 derivatives, such as albiglutide, dulaglutide, liraglutide, and semaglutide [63,64,65]. These agents are widely used clinically due to their high therapeutic potential, flexible dosing schedules (daily to weekly), and proven efficacy in reducing blood glucose and body weight in patients with T2DM.

**Figure 3:**
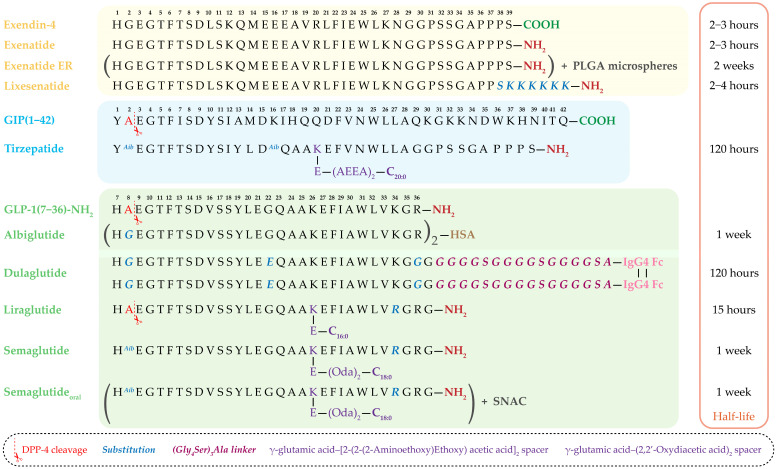
Amino acid sequences of clinically used and approved incretin-based drugs. The figure presents exendin-4–derived agents (exenatide, extended-release exenatide (ER), lixisenatide), human GLP-1 analogs (albiglutide, dulaglutide, liraglutide, semaglutide, oral semaglutide), and the dual GIP/GLP-1 receptor agonist tirzepatide. The corresponding plasma half-lives of each drug are shown on the right. Sites of DPP-4 cleavage, amino acid substitutions, and structural modifications—including fatty acid side chains, linkers, albumin- and Fc-fragment fusion—are indicated, highlighting strategies to enhance resistance to enzymatic degradation, prolong half-life, and improve pharmacokinetic properties. PLGA—Poly(lactic-co-glycolic acid), HSA—Human Serum Albumin, C16:0—Palmitic acid, C18:0—Stearic acid, C20:0—Arachidic acid, IgG4 Fc—Fc-fragment of human immunoglobulin G4, (Oda)_2_—(2,2′-Oxydiacetic acid)_2_, (AEEA)_2_—[2-(2-(2-aminoethoxy) ethoxy) acetic acid]_2_, SNAC—sodium N-[8-(2-hydroxybenzoyl)aminocaprylate].

A particularly notable advancement is the development of dual GIP/GLP-1 receptor agonists. Molecules such as tirzepatide simultaneously activate both incretin pathways, offering enhanced metabolic benefits [66,67]. The amino acid sequences of clinically approved incretin-based drugs, along with structural comparisons of exendin-4–derived agents and human GLP-1 analogs, as well as sequence modifications introduced to improve DPP-4 resistance, extend half-life, and optimize pharmacokinetics, are summarized in Figure 3.

The choice among incretin mimetics is determined by the patient’s individual characteristics, the drug’s efficacy, and the available administration regimens, dosages, and potential side effects. Some drugs, such as exenatide, have accumulated a substantial amount of clinical data. On the other hand, other newer agents may have a longer half-life and a more pronounced effect on weight loss and achieving normoglycemia. Furthermore, the cost of the medication is often an important factor when selecting a therapy.

### 3.3. Exendin-4–Based Drugs

The development of GLP-1RAs began in 1992, when J. Eng with colleagues isolated exendin-4—a peptide from the venom of the North American lizard Gila monster (Heloderma suspectum), an animal uniquely adapted to prolonged fasting [68]. Exendin-4, a 39–amino acid peptide sharing 53% homology with human GLP-1, is resistant to DPP-4 degradation due to the presence of glycine at position 2. These properties made it the prototype for the first clinically approved GLP-1RA—exenatide.

**Exenatide.** Exenatide (Byetta^®^), approved by the FDA in 2005, was the first drug in this class [69]. It is a synthetic 39–amino acid analog of exendin-4 with a C-terminal amide modification, which further improves resistance to enzymatic degradation. Exenatide reduces serum glucagon levels during hyperglycemia, suppresses appetite, and delays gastric emptying, thereby slowing the appearance of ingested glucose in the bloodstream [70]. However, its short half-life (2–4 h) necessitates twice-daily injections, and common side effects include nausea and dyspepsia. Nevertheless, clinical studies have demonstrated significant reductions in HbA1c and body weight, particularly in insulin-resistant patients. However, it also exhibited relatively high immunogenic potential, likely due to its low sequence homology with endogenous human GLP-1 [71]. Along with the need for frequent injections, this drives the development of extended-release formulations with improved safety and convenience profiles.

**Extended-release Exenatide.** Strategies to prolong exenatide activity have included chemical modifications, conjugation to macromolecules, and incorporation into sustained-release matrices. The most impactful advance in this field was the use of microspheres composed of the biodegradable poly(lactic-co-glycolic acid) (PLGA) copolymer. The resulting product, Bydureon^®^ (AstraZeneca, Cambridge, UK), approved by the FDA in 2012, provides sustained exenatide release for one week after a single injection. Lactic acid and glycolic acid—the breakdown products—are eliminated from the body as carbon dioxide and water. This formulation demonstrated superiority over several oral and injectable agents, reducing HbA1c by 1.1–2.0% and body weight by 2–4 kg over six months. Long-term use (up to six years) maintained stable glycemic control and improved cardiometabolic parameters [72,73].

**Lixisenatide.** Lixisenatide—a modified 44–amino acid exendin-4 analog—features a C-terminal polylysine extension that is linked after serine at position 39 and increases the rigidity of the molecule. Its half-life of ~1.5–4.5 h allowed once-daily dosing. Although preclinical and clinical investigations have indicated its potential neuroprotective properties, particularly in Parkinson’s disease models, the clinical application of lixisenatide since 2023 has been exclusively confined to a fixed-dose combination with insulin glargine (iGlarLixi^®^). This combination is grounded in the complementary pharmacodynamics of the two agents. The combination addresses the full 24 h glucose profile: insulin glargine, as a long-acting basal insulin, provides stable control of fasting plasma glucose, while the short-acting lixisenatide specifically targets postprandial glucose excursions through delayed gastric emptying and glucagon suppression [74].

The fixed-ratio formulation offers several clinical advantages, including once-daily administration via a single injection, which significantly improves patient convenience and treatment adherence. Furthermore, it permits the gradual, parallel titration of both components. This slow escalation of the lixisenatide dose is instrumental in mitigating the gastrointestinal adverse effects typically associated with GLP-1RAs. From an efficacy standpoint, the iGlarLixi^®^ combination demonstrates superior HbA1c reduction compared to either monotherapy while concurrently mitigating key limitations of its components—namely, the weight gain associated with insulin and the risk of hypoglycemia, achieving an optimal therapeutic balance [75,76].

### 3.4. GLP-1-Based Drugs

The development of N-terminal modifications of GLP-1RAs has become a key strategy in creating effective antidiabetic agents. The primary goal of these modifications is to enhance the molecule’s resistance to enzymatic degradation by DPP-4 while maintaining high-affinity binding to the GLP-1R and activation of its signaling cascade [77]. The most successful modifications involve substituting alanine at position 8 with glycine or α-aminoisobutyric acid (Aib8). These changes formed the basis for drugs such as semaglutide and albiglutide. Modifications at positions His7 and Glu9 have also been explored, although they have not achieved widespread clinical use [78,79].

**Liraglutide.** A prominent example of successful molecular design is liraglutide (FDA-approved in 2010 as Victoza^®^ for T2DM and in 2014 as Saxenda^®^, a GLP-1 analog with a fatty acid modification with a 16-carbon palmitic acid side chain linked by γ-glutamic acid that enables reversible binding to plasma albumin [80]. The peptide is produced recombinantly in yeast and is identical to native GLP-1 except at position 34, where lysine is replaced by arginine. Arg34 was introduced into liraglutide to enable site-specific acylation of Lys26 and had no apparent effect on binding to the GLP-1R [63]. These modifications prolong the plasma half-life of liraglutide to 13 h following subcutaneous administration, in part by facilitating its binding to plasma proteins. This allows for once-daily administration while providing robust glycemic control. Additionally, direct comparisons of liraglutide and exenatide in patients with T2DM demonstrated comparable weight loss, but liraglutide was better tolerated and produced a greater reduction in HbA1c after 26 weeks of therapy [81].

**Dulaglutide.** Another example is dulaglutide (FDA 2014, Trulicity^®^) [65,82]. It consists of two chains of GLP-1 sequences modified with a Gly8 substitution, covalently linked to the Fc-fragment of a human modified monoclonal antibody IgG4 via peptide ((Gly4Ser)3Ala) linker [83]. This linker serves several important functions. It provides flexibility and appropriate spacing between the two GLP-1 analog chains and the Fc fragment of the human IgG4 antibody. Additionally, the linker contributes to the overall stability and solubility of the molecule, facilitating prolonged circulation time and protecting against enzymatic degradation.

This design not only confers resistance to DPP-4 but also reduces immunogenicity and extends the half-life to approximately 5 days. Such prolonged biological activity allows once-weekly administration without loss of therapeutic efficacy. Two phase II clinical trials demonstrated a dose-dependent reduction in HbA1c of up to 1.52% compared with placebo. Side effects associated with dulaglutide administration were mainly gastrointestinal [82].

**Albiglutide.** Comparable pharmacokinetic properties are exhibited by albiglutide (FDA 2014, Tanzeum^®^/Eperzan^®^), which is a hybrid of two modified with Gly8 GLP-1 sequences covalently linked to human serum albumin (HSA) [64,84,85]. This provides DPP-4 resistance and prolonged action (half-life ~5 days). However, compared with dulaglutide, albiglutide demonstrates lower GLP-1R affinity, limiting its clinical use. In 2018, the drug was withdrawn from the market by the manufacturer for commercial reasons, not due to safety or efficacy concerns.

**Semaglutide (subcutaneous).** A particularly notable GLP-1RA is semaglutide (subcutaneous form FDA-approved in 2017 as Ozempic^®^ for T2DM and as Wegovy^®^ for obesity or overweight and to reduce cardiovascular events) [63]. This optimized GLP-1 analog incorporates three key modifications: an Ala8→Aib8 substitution that provides shielding from DPP-4-mediated N-terminal proteolysis; a fatty acid modification with a γ-glutamic acid-(2,2′-oxydiacetic acid)2 spacer linked to C18:0 at position 26 for stable albumin binding; and a replacement of lysine with arginine at position 34. As was the case in liraglutide, Arg34 was introduced to enable site-specific acylation at Lys26. These changes provide a half-life of up to one week, allowing for once-weekly injections [63,86]. The safety of semaglutide has been confirmed in a series of large international clinical trials, including SUSTAIN-6, which evaluated cardiovascular outcomes [86].

**Semaglutide (oral).** The PIONEER clinical trial program, comprising ten pivotal studies, evaluated the efficacy and safety of the oral formulation of semaglutide, which was approved by the FDA in 2019 under the trade name Rybelsus^®^ [87]. The oral form overcomes the limitations of peptide instability in the gastrointestinal tract through the use of the absorption enhancer SNAC (sodium N-[8-(2-hydroxybenzoyl)aminocaprylate]), which allows the half-life of oral semaglutide to reach approximately 1 week (160 h), aligning with the subcutaneously administered form [88,89]. This excipient protects the molecule from degradation in the acidic stomach environment and facilitates absorption in the small intestine. Despite the low bioavailability of the oral form (<1%), clinical efficacy is maintained due to high receptor activity. In the PIONEER program, once-daily oral administration of semaglutide at 14 mg reduced HbA1c by 1.0–1.4%.

Thus, the success of GLP-1RAs has been achieved not only through molecular modifications of the native peptide but also through the implementation of prolonged-action and oral delivery technologies. These advancements improve patient adherence, enhance clinical efficacy, and potentially reduce cardiovascular risk.

### 3.5. Dual GIP/GLP-1 Agonists

**Tirzepatide.** One of the most significant achievements in this area is tirzepatide, which was FDA-approved in 2022 as Mounjaro^®^ for T2DM and in 2023 as Zepbound^®^ for obesity [66]. Tirzepatide is a 39–amino-acid peptide modified via acylation at Lys20 with a γ-glutamic acid–[2-(2-(2-aminoethoxy)ethoxy) acetic acid]_2_ spacer ((AEEA)_2_) linked to eicosanedioic diacid (C20:0). It also contains Aib at position 2 to prevent DPP-4–mediated degradation.

Tirzepatide exhibits unbalanced agonism: high GIPR activity combined with much weaker GLP-1R activity. Nevertheless, due to high plasma concentrations and a prolonged half-life (~120 h), it achieves sustained metabolic effects, including hypoglycemic and anorexigenic actions. Despite its unbalanced receptor activity, tirzepatide demonstrated significant advantages over GLP-1RA monotherapy in clinical trials, opening new horizons for pathogenetic treatment of T2DM. Phase III clinical trials (SURPASS and SURMOUNT) showed that tirzepatide outperforms semaglutide in both HbA1c reduction and weight loss [67]. These data supported its approval first for T2DM and subsequently for obesity treatment.

Thus, the development of such GLP-1RA as dual GIP/GLP-1 agonists like tirzepatide, reflects a paradigm shift in incretin therapy—from a narrowly targeted monotherapy approach to a systemic, multi-target strategy. These drugs not only expand the therapeutic arsenal for T2DM and obesity but also pave the way for personalized medicine (see Table 1 for structural characteristics, dosing regimens, and key actions of clinically approved GLP-1RAs).

## 4. Incretins and Incretinomimetics in the Pathogenesis and Treatment of Cardiometabolic Diseases

### 4.1. Obesity and Type 2 Diabetes

Current understanding of the pathogenesis of T2DM and obesity highlights dysfunction of the incretin system as a central aspect of neuroendocrine metabolic dysregulation. Obesity is a chronic metabolic disorder characterized primarily by excessive accumulation of adipose tissue and an increased risk of cardiovascular and musculoskeletal diseases, as well as several cancers, including endometrial, breast, ovarian, and colorectal cancers [90,91,92,93,94]. The term “diabesity” reflects the strong association between obesity and T2DM, with up to 80% of T2DM patients being overweight or obese. Visceral adipose tissue in obesity is metabolically active, secreting pro-inflammatory cytokines such as TNF-α, IL-6, and free fatty acids [95]. These activate protein kinase C beta (PKCβ) and induce serine phosphorylation of IRS-1. These modifications reduce expression of GLUT-4, promote oxidative stress, and impair mitochondrial function, further contributing to insulin resistance. Moreover, obesity decreases adiponectin levels and induces leptin resistance, exacerbating metabolic imbalance. Collectively, these mechanisms drive insulin resistance (IR), the key pathogenic factor in T2DM, characterized by chronic hyperglycemia due to both IR and progressive β-cell dysfunction [96,97].

Patients with obesity and T2DM also exhibit reduced sensitivity to GLP-1 and impaired secretion of GIP, leading to insufficient glucose-dependent insulin secretion. This has spurred the development of incretin-based therapies targeting GLP-1 and GIP receptors. Under hyperglycemic conditions, GIP loses much of its insulinotropic activity due to receptor downregulation, whereas GLP-1 remains effective and can even enhance β-cell responsiveness to GIP. This principle underpins the concept of dual receptor activation [20,60]. The role of GIP in obesity is intricate and somewhat paradoxical. While GIP is implicated in pro-inflammatory and adipogenic processes that typically promote obesity, pharmacological activation of GIP receptors has been shown to exert beneficial metabolic effects, including reduced food intake, improved energy balance, and weight loss. In contrast, GLP-1RAs consistently induce weight reduction through multiple mechanisms. This apparent contradiction may be explained by differences between endogenous versus pharmacological GIP receptor activation, tissue-specific effects, or synergistic signaling interactions with other pathways such as GLP-1 [18,98].

In terms of large-scale clinical evidence in obesity management, the SURMOUNT-1 trial demonstrated that the dual GIP/GLP-1 receptor agonist tirzepatide induced substantial and sustained body-weight reductions over 72 weeks in adults with obesity or overweight without diabetes. The mean percentage change in body weight was −15.0%, −19.5%, and −20.9% with tirzepatide 5 mg, 10 mg, and 15 mg, respectively, compared with −3.1% with placebo. Notably, among secondary endpoints, 55% and 63% of participants receiving 10 mg and 15 mg achieved ≥20% weight loss, respectively [99].

Beyond treatment of T2DM, incretins are actively investigated in prediabetes and early cardiometabolic syndrome. Accordingly, GLP-1RAs therapy may increase the likelihood of reverting to normoglycemia, lower the risk of diabetes progression, and improve anthropometric and lipid profiles [59,100].

### 4.2. Cardiometabolic Health and MACE Reduction

While incretin-based therapies are widely used for glycemic control and weight reduction in patients with T2DM, it is crucial to recognize that individuals with T2DM and obesity also face a markedly elevated risk of CVDs and major adverse cardiovascular events (MACE). This heightened risk reflects the close association of T2DM and obesity with insulin resistance, a central mechanism driving both metabolic and cardiometabolic syndromes. Insulin resistance promotes sympathetic overactivation, renin–angiotensin–aldosterone system (RAAS) activation, sodium retention, and increased peripheral resistance, thereby exacerbating left ventricular dysfunction and accelerating atherogenesis. Moreover, the clustering of hyperinsulinemia, dyslipidemia (characterized by elevated triglycerides and low-density lipoprotein (LDL) alongside reduced high-density lipoprotein (HDL)), chronic inflammation, impaired nitric oxide synthesis, vasoconstriction, and oxidative stress further contributes to the pathogenesis of atherosclerosis, increasing the likelihood of ischemic heart disease and MACE such as myocardial infarction (MI) and stroke [101].

Accumulating evidence demonstrates that GLP-1RAs provide benefits extending beyond glucose lowering. Clinical trials and meta-analyses consistently report a prevention of cardiovascular events among patients with T2DM and obesity [102]. For example, GLP-1RAs significantly lower the incidence of MACE compared with placebo, with meta-regression analyses suggesting an approximate 25% relative risk reduction per 1% decrease in HbA1c, to which weight loss and anti-inflammatory effects also contribute [103,104].

Semaglutide, one of the most extensively studied GLP-1RAs, has shown particular promise. In a meta-analysis of arrhythmic outcomes, semaglutide therapy significantly reduced the risk of atrial fibrillation, although no significant effects were observed for atrial flutter, ventricular tachycardia, supraventricular tachycardia, ventricular fibrillation, or sinus node dysfunction. Regarding atrioventricular (AV) conduction disorders, semaglutide reduced the risk of complete AV block but did not significantly affect first- or second-degree AV block. Importantly, semaglutide was associated with a significant reduction in cardiovascular mortality and the need for revascularization, though no clear effects were detected on the risk of non-fatal MI, non-fatal stroke, unstable angina, or heart failure [105]. Moreover, both semaglutide and liraglutide have demonstrated cardiovascular risk reduction in patients with T2DM. In the landmark LEADER and SUSTAIN-6 cardiovascular outcome trials (CVOTs), these drugs reduced the incidence of MI, stroke, and cardiovascular death by 12–26% [106,107]. The SELECT trial, involving over 17,000 adults with overweight or obesity and established cardiovascular disease but without diabetes, showed that once-weekly semaglutide (2.4 mg) reduced the risk of MACE, such as cardiovascular death, nonfatal MI, or nonfatal stroke, by 20% compared with placebo [108].

At the same time, the SOUL trial demonstrated that oral semaglutide reduced the composite outcome of cardiovascular death, nonfatal MI, or nonfatal stroke by 14% (HR 0.86; 95% CI 0.77–0.96) in high-risk patients with T2DM and established atherosclerotic cardiovascular disease (ASCVD) and/or chronic kidney disease (CKD) [109]. 

Furthermore, the SURPASS-CVOT trial confirmed the cardiovascular safety of tirzepatide, meeting the primary endpoint of non-inferiority for MACE-3 (cardiovascular death, MI, or stroke) versus dulaglutide (HR 0.92; 95.3% CI 0.83–1.01) while also demonstrating a 16% reduction in all-cause mortality (HR 0.84; 95% CI 0.75–0.94) [110]. 

Beyond arrhythmic and macrovascular outcomes, semaglutide demonstrates potential benefits for microvascular complications. While no significant effect was observed on the risk of diabetic retinopathy progression, a favorable, albeit non-significant, trend was noted toward reducing new-onset or progressive nephropathy. These findings suggest a clinically meaningful direction, emphasizing the need for further trials to clarify the nephroprotective effects of semaglutide [105].

Additional studies reinforce the cardioprotective profile of semaglutide in patients with T2DM. Subcutaneous administration was associated with substantial reductions in the risk of hospitalization due to heart failure, cardiovascular mortality, all-cause mortality, non-fatal MI, coronary revascularization, and stroke. In another meta-analysis of 11 CVOTs with 82,140 participants, GLP-1RAs demonstrated a 16% relative reduction in stroke risk compared to placebo [103]. Nevertheless, treatment was accompanied by a higher relative risk of certain adverse effects, underscoring the need for careful patient selection and monitoring [111].

### 4.3. MASLD/MASH: The Hepatic Component

The liver represents another major target organ affected by metabolic disturbances, particularly in the context of obesity and insulin resistance. The global prevalence of metabolic dysfunction-associated steatotic liver disease (MASLD), previously termed non-alcoholic fatty liver disease (NAFLD), is estimated at 32.4%, underscoring its rising clinical significance [112]. MASLD encompasses both simple steatosis and its progressive form, metabolic dysfunction-associated steatohepatitis (MASH). Both entities are strongly linked to insulin resistance, obesity, and T2DM, and are associated with increased risks of liver fibrosis progression and liver-related as well as all-cause mortality.

Human hepatocytes express GLP-1Rs, and their activation by GLP-1RAs is hypothesized to exert beneficial effects on hepatic lipid metabolism. These include reductions in steatosis, enhancement in fatty acid oxidation, attenuation of lipotoxicity, and modulation of pro-inflammatory cytokines implicated in fibrogenesis [113]. Accordingly, GLP-1RAs have increasingly been recognized as promising therapeutic agents for MASLD, acting both indirectly via improvements in glycemic control, reduced glucagon secretion, delayed gastric emptying, and significant weight loss, and directly on hepatic metabolic pathways. Evidence indicates that agents such as semaglutide and liraglutide decrease hepatic steatosis, improve insulin sensitivity, and mitigate hepatic inflammation [114,115].

A meta-analysis evaluating 24 weeks of semaglutide treatment in patients with NAFLD or NASH demonstrated significant reductions in alanine aminotransferase (ALT) and aspartate aminotransferase (AST), decreased hepatic fat content and stiffness, and improvements in HbA1c and lipid profiles. Collectively, these findings suggest that GLP-1RAs address key pathophysiological mechanisms of MASLD/MASH, offering both metabolic and hepatic benefits. Nevertheless, gastrointestinal adverse effects, including nausea, vomiting, dyspepsia, appetite loss, constipation, and diarrhea, as well as gallbladder disorders were frequently reported, highlighting the importance of tolerability considerations in long-term therapy [116].

Randomized controlled trials have further substantiated the efficacy of GLP-1RAs in MASH [117]. In the LEAN study, 39% of patients receiving liraglutide achieved histological resolution of MASH without fibrosis progression, while in the SEMA-NASH trial, up to 59% of patients treated with semaglutide (0.4 mg) achieved similar outcomes [118]. These improvements were accompanied by slower fibrosis progression and favorable changes in non-invasive markers such as transient elastography and enhanced liver fibrosis scores. However, significant histological regression of fibrosis was not observed, likely due to the relatively short duration of therapy. Studies with exenatide also demonstrated reductions in hepatic fat content, body weight, transaminases, and FIB-4 index, alongside improvements in lipid profiles, further confirming the mechanistic role of GLP-1RAs in modulating MASLD/MASH [119]. In the ESSENCE trial, once-weekly semaglutide (2.4 mg) achieved resolution of steatohepatitis without worsening of fibrosis in 62.9% of participants compared with 34.3% in the placebo group after 72 weeks. Improvement in liver fibrosis (≥1 stage) without worsening of steatohepatitis occurred in 36.8% versus 22.4%, respectively [120].

Beyond monotherapy, dual agonists targeting GLP-1 and other metabolic pathways have shown even greater therapeutic potential. Agents such as tirzepatide and GLP-1/glucagon co-agonists (cotadutide, pemvidutide, BI 456906/survodutide) have produced superior outcomes in reducing hepatic fat, improving insulin resistance, and attenuating lipotoxicity and inflammation [115]. For instance, cotadutide reduced hepatic fat by 33% and decreased markers such as transaminases and PRO-C3, while pemvidutide achieved >90% hepatic fat reduction within 6 weeks. In the SURPASS-3 substudy, tirzepatide reduced hepatic fat by 8.1% over 52 weeks, accompanied by reductions in ALT, AST, and γ-GT [121]. The pronounced weight loss observed with these agents likely represents the primary driver of steatosis reversal and fibrosis attenuation. These advances have catalyzed the development of triple agonists (GLP-1/GIP/glucagon), such as retatrutide, which may further amplify effects on body weight and metabolic outcomes.

Similarly, the SYNERGY-NASH phase 2 trial evaluated tirzepatide in patients with nonalcoholic steatohepatitis (NASH). After 52 weeks, 51.8%, 62.8%, and 73.3% of participants treated with tirzepatide 5 mg, 10 mg, and 15 mg, respectively, achieved resolution of MASH without fibrosis worsening, compared with 13.2% in the placebo group. For fibrosis improvement without worsening of MASH, the corresponding rates at the secondary endpoint were 59.1%, 53.3%, and 54.2% versus 32.8% with placebo [122].

Taken together, GLP-1RAs and next-generation incretin-based therapies target fundamental mechanisms underlying MASLD and MASH. Their ability to simultaneously modulate glucose homeostasis, body weight, hepatic lipid metabolism, and inflammation positions them as highly promising therapeutic strategies. Ongoing studies will clarify their long-term efficacy and safety profiles, as well as their potential integration into combinatorial treatment regimens aimed at improving clinical outcomes in patients with MASLD/MASH.

### 4.4. Kidney Protection

Incretin receptors, particularly GLP-1R, are also expressed in the kidneys and activation of GLP-1R has been associated with reduced incidence of renal impairment [123].

Chronic kidney disease (CKD) arises from progressive injury to renal structures, primarily glomeruli, tubulointerstitium, and vasculature, leading to impaired filtration, sodium retention, endothelial dysfunction, and activation of neurohormonal pathways such as the RAAS. Metabolic disorders, including obesity, insulin resistance, dyslipidemia, and systemic inflammation, exacerbate these pathological processes, establishing a bidirectional link between CKD and cardiometabolic disease [124]. Among the etiologies of CKD, T2DM remains the predominant cause worldwide. Diabetic nephropathy (DN), a major microvascular complication of T2DM, is characterized by structural and functional renal alterations, including glomerular basement membrane thickening, mesangial expansion, and podocyte loss. Clinically, DN manifests as persistent albuminuria and a progressive decline in glomerular filtration rate (GFR). Its pathogenesis involves chronic exposure to metabolic insults (hyperglycemia, insulin resistance, obesity), hemodynamic stress (glomerular hyperfiltration, intraglomerular hypertension, RAAS activation), and inflammatory-fibrotic mediators such as oxidative stress, pro-inflammatory cytokines, and immune pathway activation [125].

Conventional management of diabetic kidney disease (DKD) primarily targets RAAS inhibition to reduce intraglomerular pressure and slow disease progression. However, growing evidence indicates that GLP-1RAs exert additional nephroprotective effects beyond glycemic control. Large phase III cardiovascular outcome trials have demonstrated that GLP-1RAs significantly reduce the incidence of new-onset macroalbuminuria and slow the rate of GFR decline in patients with T2DM, highlighting renal benefits partially independent of glucose lowering [123,126].

Importantly, clinical studies suggest that only 10–25% of these nephroprotective effects are attributable to improvements in glycemia, body weight, and blood pressure. For instance, in the AWARD-7 trial, dulaglutide significantly attenuated GFR decline compared with insulin glargine, despite similar control of glycemia and blood pressure in both arms [127]. These findings implicate additional mechanisms, including anti-inflammatory and antioxidant actions, supported by reductions in IL-6, MCP-1, and TNF-α levels, decreased immune cell infiltration into renal tissue, and inhibition of fibrotic remodeling [128].

The recently published FLOW trial provided the first definitive evidence of renoprotective efficacy of semaglutide in patients with T2DM and CKD. Over a median follow-up of 3.4 years, once-weekly semaglutide (1.0 mg) reduced the risk of the composite primary endpoint—comprising kidney failure, a sustained ≥50% decline in eGFR, or death from kidney or cardiovascular causes—by 24% compared with placebo (HR 0.76; 95% CI 0.66–0.88; *p* < 0.001) [129]. This landmark study establishes GLP-1RAs as therapeutic agents capable of modifying renal outcomes beyond glycemic control.

At the molecular level, recent research has identified several emerging targets that may interact with GLP-1RA signaling in mitigating DKD progression. These include the NLRP3 inflammasome, oxidative stress–related pathways (e.g., NRF2, SIRT1, and AMPK), and regulators of renal lipid metabolism and fibrosis, such as TGF-β1/Smad and Wnt/β-catenin signaling [130,131]. Targeting these molecular pathways, in combination with incretin-based therapies, represents a promising strategy for preventing renal function decline in patients with diabetes and CKD.

Given that kidney protection remains a central therapeutic objective in T2DM management, GLP-1RAs have emerged as a valuable therapeutic option for patients with T2DM and CKD, especially those with coexisting obesity or elevated cardiovascular risk. Their efficacy persists even in moderate-to-severe CKD, underscoring their potential as an integral component of comprehensive cardiometabolic care. Further research is warranted to refine our understanding of their nephroprotective mechanisms and long-term clinical benefits [123]. Thus, a growing body of clinical evidence highlights the multifaceted benefits of GLP-1RAs and next-generation incretin-based therapies across cardio–renal–metabolic diseases. Numerous large-scale randomized controlled trials have demonstrated reductions in CVDs, including MACE, ASCVD and heart failure with preserved ejection fraction (HFpEF), improvements in renal outcomes, and potential benefits in MASH and MASLD. Table 2 summarizes key clinical trials evaluating incretin-based agents across a spectrum of cardiometabolic and renal disorders, reflecting the rapid evolution of this therapeutic class and its expanding indications.

### 4.5. Beyond Glycemia

Beyond metabolic regulation, incretin-based therapies exhibit a wide range of pleiotropic effects, with their therapeutic potential expanding into fields such as neurocognitive disorders, Parkinson’s disease, knee osteoarthritis, chronic pain, polycystic ovary syndrome, substance use disorders, and obstructive sleep apnea [34]. GLP-1RAs act at the crossroads of metabolic, inflammatory, and neuroendocrine pathways, reflecting their expression in multiple tissues, including the CNS, cardiovascular system, and immune cells [132].

A key area of expanding clinical utility is neuroprotection. Preclinical and clinical studies suggest that GLP-1RAs provide neuroprotective benefits through multiple mechanisms, including reducing neuroinflammation, lowering oxidative stress, improving mitochondrial function, and modulating β-amyloid and tau pathology [91,133]. These effects contribute to a lower risk of neurodegenerative and neuropsychiatric disorders, including Alzheimer’s disease, dementia, Parkinson’s disease [134]. GLP-1RAs have also shown anticonvulsant properties, suggesting potential applications in epilepsy [135]. Notably, semaglutide has been reported to reduce alcohol craving in patients with obesity, indicating potential use in treating addictive disorders [133].

Importantly, multi-agonist incretin mimetics may surpass conventional GLP-1RAs. Dual (GLP-1/GIP) and triple (GLP-1/GIP/glucagon) receptor agonists have demonstrated enhanced neuroprotective efficacy in preclinical models, showing superior effects on neuronal survival, mitochondrial function, and synaptic plasticity [136,137]. Early translational studies suggest that such therapies may improve memory, reduce tau phosphorylation, and decrease β-amyloid plaque burden more effectively than monotherapy, paving the way for clinical evaluation in Alzheimer’s and Parkinson’s disease. Tirzepatide and other next-generation incretin mimetics are now under investigation for cognitive and neuropsychiatric endpoints.

The underlying molecular mechanisms—suppression of pro-inflammatory cytokines, reduction in oxidative stress, and improvement in mitochondrial function—also confer broader systemic benefits. GLP-1RAs modulate immune responses across multiple organ systems, providing protection in conditions characterized by chronic inflammation. Clinical and experimental studies demonstrate their ability to reduce the incidence and severity of severe infections and sepsis, attenuate liver and intestinal inflammation, slow atherosclerosis progression, and ameliorate autoimmune disorders. Beneficial effects have also been observed in respiratory diseases, including chronic obstructive pulmonary disease (COPD) and pneumonia [138,139,140,141].

Such pleiotropic actions support the concept of incretin-based therapy as a multimodal intervention, particularly valuable in multimorbid conditions where metabolic, inflammatory, and neurodegenerative processes intersect. This rationale has spurred growing interest in their potential role in complex syndromes such as COVID-19 and post-COVID syndrome, where metabolic dysregulation, systemic inflammation, and neurocognitive impairments coexist [142,143].

## 5. Comparative Therapeutic Perspectives: Integrative Role of Incretins

Although bariatric surgery is widely regarded as the most efficacious and enduring treatment for severe obesity, resulting in dramatic weight loss (60–80% excess weight loss), significant improvement in comorbidities, and reduced long-term healthcare costs, it carries a substantial burden of complications and concerns [144]. Key complications include nutrient deficiencies (iron, vitamin B12, calcium, vitamin D, and protein), which can lead to anemia, bone demineralization, and alopecia. Gastrointestinal pathologies are common and can involve anastomotic leaks, strictures, marginal ulcers, internal hernias, bowel obstructions, and gastrointestinal hemorrhage. Specific to banded procedures are risks of gastric slippage, band erosion, and pouch dilation. Other serious concerns include gallstone formation from rapid weight loss, postprandial hypoglycemia, and dumping syndrome. From a psychosocial perspective, patients require thorough pre-surgical psychological evaluation, as psychopathology (e.g., depression, anxiety, binge eating disorder) is common; while not an absolute contraindication, untreated severe psychiatric disorders or active substance abuse are major concerns. Postoperatively, patients must adapt to massive lifestyle changes, and there is a risk for the reemergence of disordered eating patterns, such as binge eating, which can lead to weight regain. Finally, while mortality rates have improved, perioperative risk remains, influenced by factors like advanced age, male gender, and surgeon inexperience [145,146]. Given these significant drawbacks and the inherent limitations of surgical intervention, there is a pressing need for effective non-surgical therapeutic alternatives.

**Phentermine and phentermine**/**topiramate (PHEN/TPM).** One non-surgical approach to treating obesity involves targeting the appetite center in the CNS. Phentermine, a sympathomimetic amine, acts as an appetite suppressant by predominantly increasing levels of norepinephrine, dopamine, and serotonin in the hypothalamus. Recent estimates of short-term weight loss (over 8 to 12 weeks) with phentermine monotherapy range from 3.6% to 12.1% of initial body weight [147].

The combination of PHEN/TPM represents a more potent anti-obesity medication. In this combination, topiramate promotes satiety through neuro-stabilizing properties. This therapy results in robust, dose-dependent weight loss, with an average reduction of 7.73 kg compared to placebo and higher rates of clinically significant (≥5%, ≥10%) weight loss [148].

However, the therapeutic profile of these centrally acting agents is primarily centered on weight loss and metabolic improvements (e.g., reduced waist circumference, blood pressure, and lipid levels), with less established cardio-renal benefits compared to GLP-1RAs. Furthermore, while phentermine monotherapy is generally well-tolerated—with large-scale observational studies showing no increased risk of cardiovascular events, significant blood pressure elevations, or evidence of addiction—the PHEN/TPM combination is associated with a more significant side effect profile. This includes paresthesia, dry mouth, dysgeusia, cognitive effects, and potential teratogenicity, necessitating careful patient selection and monitoring [148]. Despite being commonly prescribed anti-obesity medications, the lack of more extensive long-term safety data poses a challenge for the chronic management of patients. Thus, while PHEN/TPM is highly effective for weight loss, its role is complementary to incretins, which offer a broader, organ-protective strategy for high-risk patients.

In this context, incretin-based therapies offer a promising non-surgical approach, occupying a unique position among pharmacological agents targeting the cardio–renal–metabolic syndrome (CRMS) due to their multifaceted actions on glucose metabolism, adiposity, cardiovascular health, renal function, and systemic inflammation. Unlike traditional antidiabetic and anti-obesity agents, incretins deliver comprehensive metabolic benefits, including potent glucose-lowering efficacy, sustained weight reduction, blood pressure modulation, anti-inflammatory, and antioxidant effects, as well as emerging neuroprotective properties. In the management of CRMS, several pharmacological classes are employed, including metformin, RAAS inhibitors, statins, and sodium–glucose cotransporter 2 inhibitors (SGLT2i), each targeting distinct yet interconnected pathophysiological pathways.

**Metformin.** As the first-line therapy for T2DM, metformin primarily acts by enhancing insulin sensitivity, suppressing hepatic gluconeogenesis, and improving peripheral glucose utilization. These effects contribute to glycemic control and modest weight stabilization. However, its cardiovascular benefits remain limited, and its use is contraindicated in advanced CKD due to the risk of lactic acidosis. In contrast, GLP-1RAs preserve efficacy and safety even in moderate-to-severe CKD, addressing unmet needs in this patient subgroup. Therefore, metformin provides a metabolic foundation, while incretins complement it with cardiovascular and renal protection [149,150].

**RAAS inhibitors.** RAAS inhibitors, including ACE inhibitors and angiotensin receptor blockers, remain the cornerstone of nephroprotection in T2DM by mitigating glomerular hypertension, proteinuria, and fibrosis. However, they lack effects on glycemic control, body weight, and lipid metabolism. When combined with incretin-based therapies, RAAS inhibitors provide synergistic renal protection, integrating hemodynamic stabilization with metabolic and anti-inflammatory modulation [151]. Also, there is growing evidence that incretins have modulatory effects on renin-angiotensin system activity. Thereby, they can be promising therapeutic agents for the management of renal and cardiovascular disorders. But the exact molecular interactions between incretins and renin-angiotensin system are not clearly understood [152].

**Statins.** Statins are indispensable for ASCVD prevention, especially when there is high adherence [153]. By reducing LDL by approximately 30–50% for different drugs and lowering the atherosclerotic plaque burden, they reduce major cardiovascular events [154]. Although statins address dyslipidemia, they do not impact glycemia, weight, or inflammatory tone to the same degree. Co-administration with incretins can probably provide additive cardiometabolic benefits, targeting both lipid-driven and metabolism-driven pathways of cardiovascular risk [155].

**SGLT2i.** SGLT2i represent another pivotal class with cardio–renal benefits. By promoting glycosuria, they achieve moderate glucose lowering while reducing intraglomerular pressure and ameliorating hyperfiltration, which are key mechanisms in nephroprotection [156]. SGLT2i also confer robust heart failure protection, particularly in heart failure with reduced ejection fraction, and modest weight loss [157]. However, unlike incretins, they have limited effects on appetite regulation, satiety, and neurocognitive outcomes. Their mechanisms are primarily hemodynamic and renal, whereas GLP-1RAs engage central and peripheral pathways involving metabolism, inflammation, and oxidative stress.

Meta-analyses confirm that both GLP-1RAs and SGLT2i significantly reduce systolic and diastolic blood pressure, with weight reduction strongly correlated with blood pressure improvement in GLP-1RA-treated patients. Moreover, SGLT2i attenuates hepatic steatosis, while GLP-1RAs exert more pronounced effects on visceral adiposity and postprandial lipemia. Emerging evidence indicates sex-specific cardiovascular outcomes, with greater relative risk reduction in women treated with GLP-1RAs compared with men [158].

Taken together, incretins offer a multidimensional therapeutic platform that cannot be replicated by single-target agents. Their combination with metformin, SGLT2 inhibitors, RAAS blockers, and statins constitutes a comprehensive, organ-protective strategy addressing the metabolic, hemodynamic, inflammatory, and lipid components of CRMS. Such holistic integration positions incretin-based therapies as a cornerstone of modern cardio–renal–metabolic management, capable of reducing residual risk and improving long-term outcomes in patients with T2DM, obesity, and high cardiovascular and renal burden.

## 6. Adverse Events Associated with GLP-1RA Therapy

Although the addition of GLP-1RAs to therapy reduces the risk of adverse outcomes by approximately 24%, deterioration occurs in 10.86% of cases, and no statistically significant changes are observed in 65.14%. One of the main limitations of this drug class remains side effects, primarily affecting the gastrointestinal tract [159]. Gastrointestinal disorders occur in up to 70% of patients and are exacerbated by dose escalation. Therefore, treatment with incretin mimetics is usually initiated with low doses and gradually increased to reduce the risk of gastrointestinal side effects. The most common symptoms are nausea, vomiting, diarrhea, decreased appetite, abdominal pain, dyspepsia, and constipation. Among GLP-1RA drugs, the most pronounced nausea, vomiting, and constipation are associated with semaglutide, whereas liraglutide more often causes upper abdominal pain. These symptoms are usually most intense during the first four weeks of therapy or after a sudden dose increase. Their development mechanism is related to delayed gastric emptying and effects on appetite regulation centers. In rare cases, severe vomiting or diarrhea may lead to dehydration and arterial hypotension [160,161,162].

A serious concern is the potential link between GLP-1RA therapy and the development of pancreatitis [163,164]. This phenomenon was first described in animal studies, where exenatide caused chronic pancreatic changes in rats [165]. The proposed mechanism involves hyperplasia of beta cells and exocrine ducts, increasing organ mass and narrowing its ducts, which raises the risk of obstruction and inflammation. An analysis of 2313 reports of pancreatitis associated with hypoglycemic agents found a predominant link with GLP-1RAs compared to DPP-4 inhibitors and sodium-glucose cotransporter 2 (SGLT2) inhibitors. Additionally, liraglutide showed the strongest association with pancreatitis [166].

Possible complications such as intestinal obstruction and gastroparesis have also been reported, primarily with long-term use. Some patients develop gallbladder pathology such as cholelithiasis and cholecystitis most often after ≥26 weeks of therapy, especially with rapid and significant weight loss [167].

Allergic reactions are relatively rare but require attention, especially with long-term use. The most common are local injection-site hypersensitivity reactions–itching, redness, swelling, that usually resolve spontaneously. In rare cases, particularly with exenatide and lixisenatide, generalized reactions may occur: urticaria, angioedema, psoriasis-like eruptions, and pustular rashes. Anaphylaxis is extremely rare but requires immediate intervention [162,168].

Renal complications have included acute kidney injury, mainly due to hypovolemia from vomiting, diarrhea, or reduced fluid intake. Patients with hypertension, heart failure, or those taking angiotensin-converting enzyme (ACE) inhibitors or angiotensin II receptor blockers are at higher risk. Interstitial nephritis and tubular necrosis have also been reported, particularly with exenatide and liraglutide therapy. Metabolic complications include hypoglycemia, especially when GLP-1RAs are combined with insulin or sulfonylureas, requiring careful dose adjustment. Monotherapy with GLP-1RAs or combination with metformin generally does not lead to hypoglycemia. Rare cases of diabetic ketoacidosis have been reported, particularly after rapid insulin withdrawal [61].

Ophthalmologic complications are also important. Some studies show an increased risk of diabetic retinopathy progression, especially in patients with preexisting changes and rapid HbA1c reduction. These effects are mainly associated with injectable forms of semaglutide, exenatide, and dulaglutide, whereas oral semaglutide has not shown a similar risk [169,170]. Moreover, concerns have been raised regarding the occurrence of non-arteritic anterior ischemic optic neuropathy (NAION) in some patients. A retrospective study demonstrated an increased risk of NAION in diabetic patients taking semaglutide [171]. However, another study found no statistically significant increase in NAION risk associated with semaglutide use [172]. These conflicting results may be explained by the limitations of these studies, including short follow-up periods, lack of adjustment for potential confounding factors, insufficient ethnic diversity, and relatively small sample sizes (710 and 18,657 T2DM patients, respectively). In contrast, a much larger and more rigorously designed study involving 174,584 T2DM patients of diverse ethnic backgrounds taking semaglutide identified an increased risk of NAION after 2 years of treatment [173]. Nevertheless, further in-depth studies are needed to confirm the potential impact of GLP-1RA on NAION development.

Emerging evidence has also raised questions regarding potential neuropsychiatric effects associated with GLP-1RA therapy. Recent pharmacovigilance analyses and observational studies have reported isolated cases of depression, anxiety, and suicidal ideation among patients receiving GLP-1RAs, particularly in the context of obesity management rather than diabetes treatment. Although causality remains unproven and randomized clinical trials have not demonstrated a significant increase in psychiatric adverse events, population-based evidence warrants attention. In a large cohort study, Kornelius et al. found that GLP-1RA treatment was associated with a 98% increased risk of any psychiatric disorder, including a 195% higher risk of major depression, 108% higher risk of anxiety, and 106% higher risk of suicidal behavior among patients with obesity [174]. These findings underscore the importance of careful psychiatric assessment before and during GLP-1RA therapy, particularly in individuals with preexisting mood disorders, and highlight the urgent need for prospective clinical trials to better clarify the causal relationship and underlying neurobiological mechanisms.

## 7. Future Prospects

The study of incretins spans over a century and reflects the evolving understanding of hormonal regulation of digestion, metabolism, and diabetes pathogenesis. Since the first observations in the early 20th century, numerous researchers have contributed to the development of incretin theory, culminating in the creation of effective drugs for modern metabolic disease therapy. The significance of incretin-based therapies is increasing in light of the global rise in high-calorie diets and sedentary lifestyles, especially among socially vulnerable populations with limited access to balanced and healthy nutrition [175]. This environment promotes maladaptive eating patterns, including rational overeating, where cycles of excessive caloric intake alternate with restrictive behaviors, exacerbating insulin resistance, visceral adiposity, and lipid dysregulation [176]. Given the multifactorial etiology of obesity, including behavioral, neuroendocrine, genetic, and socio-economic factors, pharmacological interventions alone cannot provide a complete solution. Nonetheless, incretin-based agents, by modulating central appetite-regulating circuits and peripheral energy metabolism, have been recognized as a pivotal component of multimodal treatment strategies as part of integrated lifestyle and psychosocial interventions.

Clinical application of incretin therapy is rapidly evolving, shifting from glucose-centric management of T2DM toward a multidimensional strategy addressing cardiovascular, metabolic, and neurobehavioral comorbidities. Current clinical evidence robustly supports the cardioprotective potential of GLP-1RAs, as they mitigate MACE, reduce fibrosis, improve endothelial function and vascular remodeling and continue to demonstrate robust efficacy and safety across multiple large-scale clinical trials and CVOTs, including LEADER (liraglutide), SUSTAIN-6 and PIONEER-6 (semaglutide), REWIND (dulaglutide), EXSCEL (exenatide), HARMONY (albiglutide), SOUL (oral semaglutide), SURPASS-CVOT (tirzepatide), and FLOW (semaglutide in CKD). Collectively, these studies reinforce the cardiometabolic, renal, and potentially neuroprotective benefits of incretin-based therapies, extending their relevance well beyond glycemic control. However, incomplete understanding of underlying mechanisms underscores the necessity for further mechanistic research to delineate GLP-1R- and GIPR-dependent and independent pathways. Clarifying these tissue-specific signaling cascades, including β-arrestin-mediated and biased agonist effects, may facilitate the development of next-generation incretin mimetics with enhanced efficacy and safety.

Notably, the therapeutic application is expanding toward broader and even off-label applications, reflecting the global impact of metabolic dysregulation across organ systems. As obesity and insulin resistance drive multisystem pathology, GLP-1RAs and related incretin-based drugs are increasingly relevant across diverse medical specialties. This represents a paradigm shift in clinical management, moving the treatment of obesity from the exclusive domain of endocrinology into the hands of cardiologists, nephrologists, hepatologists, and neurologists. Armed with robust evidence, these specialists can now directly target obesity as a root cause of the conditions they treat, fundamentally expanding their therapeutic arsenal beyond conventional, often symptomatic, interventions.

Nevertheless, while these agents show extraordinary promise, longer-term data, real-world evidence, and continued pharmacovigilance are essential to fully understand their efficacy, safety, and cost-effectiveness across heterogeneous populations. Future clinical integration of incretin therapies should be guided by careful patient selection, gradual expansion of indications, and interdisciplinary collaboration, ensuring that their transformative potential is realized safely and sustainably.

Despite their clinical promise, current incretin-based therapies face several limitations, including tolerance development, gastrointestinal side effects, potential risks for pancreatic and neurological adverse events, the need for long-term administration to sustain benefits, and the partial reversal of metabolic improvements upon discontinuation [160,177]. Furthermore, chronic overstimulation of the brain’s reward pathways in modern food environments undermines long-term adherence and efficacy, indicating that pharmacotherapy must be complemented by behavioral, educational, and environmental interventions [178,179,180].

A critical technological challenge lies in the delivery route. Owing to their peptide nature, GLP-1RAs exhibit low oral bioavailability. Oral peptide administration remains limited by rapid gastrointestinal degradation, reducing oral bioavailability and clinical efficacy. According to Lipinski’s Rule of Five (Ro5), orally active compounds are limited in hydrogen bonding capacity, molecular weight, and lipophilicity, criteria often violated by therapeutic peptides [181]. As a result, injectable formulations remain the predominant mode of administration. Innovative strategies, including peptide cyclization, incorporation of noncanonical amino acids, terminal group modifications, and nanocarrier-based delivery systems, are being developed to enhance stability and gastrointestinal absorption [182]. Despite technological advances, the absence of universal design principles for compounds beyond Ro5 criteria highlights the need for systematic studies of physicochemical and transport properties to guide rational oral peptide drug development.

In addition, despite its advantages, chemical synthesis has notable constraints. It is generally feasible only for sequences up to ~50 residues, as longer chains result in reduced yields and increased side-product formation. For longer peptides, hybrid approaches combining chemical and biological methods are used, though these increase production complexity and cost [58]. Purification can also be challenging due to the formation of epimers, deletion sequences, and other by-products, often requiring high-performance chromatography [183]. Additional technical issues include poor scalability of certain reactions, such as disulfide bond formation, and the potential immunogenicity of synthetic modification fragments, such as PEG, when used chronically [53,184]. These challenges also need to be overcome for more efficient and less costly drug production.

Parallel to chemical and nanotechnological innovations, biological strategies are also emerging. Recent advances in microbial biotechnology have introduced genetically engineered probiotic strains as living delivery systems for incretin-based therapies. Recombinant bacteria, including Lactobacillus and Escherichia coli, engineered to secrete GLP-1 or its analogues, have demonstrated efficacy in improving glucose tolerance, lipid metabolism, and insulin sensitivity in diet-induced obese and diabetic rodent models. These bioengineered microbial platforms offer a promising strategy for oral GLP-1RA delivery by protecting the peptide from gastrointestinal degradation, enhancing pancreatic targeting, and simultaneously modulating gut microbiota composition, thereby integrating metabolic and microbial benefits into a unified therapeutic approach [185,186].

While technological innovation advances drug delivery, global implementation faces barriers of cost, production, and equitable access. Implementation research should therefore prioritize equitable access, cost-effectiveness analyses, and healthcare provider education to ensure that the benefits of incretin-based therapies reach high-risk and underserved populations. In addition to formulation challenges, production capacity, and supply limitations have led to intermittent global shortages of GLP-1RAs, underscoring the need for strategic allocation based on clinical urgency and health equity principles. Addressing these limitations requires both expansion of manufacturing infrastructure and priority-based allocation frameworks to ensure timely access for patients at highest risk of premature morbidity and mortality [187].

Looking forward, the next generation of incretin-based therapies is likely to rely on multi-agonist approaches (e.g., GLP-1/GIP, GLP-1/glucagon, and triple agonists) that integrate complementary metabolic and neurohormonal effects, achieving superior efficacy in weight reduction, insulin sensitivity, and organ protection. Dual and triple agonists (e.g., tirzepatide, cotadutide, retatrutide) have demonstrated unprecedented weight reduction and metabolic improvements with ongoing trials expected to clarify their long-term benefits. Such multi-target approaches may overcome limitations of monotherapy, reducing required doses and mitigating tolerance or gastrointestinal side effects. Additionally, combination regimens pairing incretins with SGLT2 inhibitors, lipid-lowering agents, or anti-inflammatory compounds hold promise for synergistic management of CRMS. Emerging technologies, including digital therapeutics and wearable biosensors, may further personalize therapy, optimizing dosing schedules and enhancing adherence.

In summary, the future of incretin-based therapy lies in a multi-level approach encompassing mechanistic elucidation of receptor signaling and tissue-specific effects, clinical expansion into cardiovascular and neurological indications, technological advancements enabling novel delivery systems and oral formulations, and systemic implementation strategies integrating pharmacological, behavioral, and societal dimensions. Such integrative approaches and collaborative strategies are essential to address the multifaceted pathophysiology of metabolic disorders and to maximize long-term clinical and societal benefits.

## 8. Conclusions

Incretins have been the subject of intense scientific investigation for several decades, culminating in the development of GLP-1RAs, which have revolutionized the treatment landscape for metabolic diseases. These incretin mimetics have transcended their original role in glycemic control, demonstrating impressive efficacy not only in lowering blood glucose levels but also in facilitating significant weight loss and improving cardiometabolic outcomes, including reduction of MACE. Moreover, GLP-1RAs have shown beneficial effects on hepatic and renal complications associated with metabolic dysregulation.

Beyond metabolic disorders, the therapeutic potential of GLP-1RAs continues to extend into the treatment of neurodegenerative diseases, orthopedics, pain management, endocrine disorders, and psychiatry. Nevertheless, the widespread adoption of GLP-1RAs remains challenged by notable adverse effects, predominantly gastrointestinal intolerance, and unresolved long-term safety concerns including pancreatic, renal, and ophthalmologic risks. Another clinical hurdle is the rapid weight regain often observed upon discontinuation of therapy if lifestyle and dietary modifications are not concurrently implemented.

To maximize patient adherence and broaden accessibility, ongoing innovation in drug development is crucial. This includes the creation of novel formulations to prolong drug action, as well as alternative peptide production methods that reduce dependency on costly chemical synthesis. Such advances will be pivotal in lowering treatment costs, enhancing drug availability, and addressing the global obesity epidemic more effectively.

In summary, GLP-1RAs represent a transformative breakthrough in metabolic disease management, with a rapidly expanding spectrum of therapeutic applications. Their full potential is being actively defined by numerous phase 3 and real-world studies, which are evaluating long-term outcomes in cardiovascular, renal, and neurocognitive health, as well as benefits in non-traditional indications like NASH and neurodegenerative disorders. As this robust evidence base matures, the key challenges will be to optimize their long-term safety and ensure global accessibility.

## Figures and Tables

**Table 1:** Characteristics of clinically approved GLP-1RAs.

Drug	Structural Features	Dosing Regimen
Exenatide (Byetta^®^)	Exendin-4–based, C-terminal amidation	Twice daily
Extended-release Exenatide (Bydureon^®^)	Exendin-4–based, C-terminal amidation, encapsulation in PLGA copolymer microspheres	Once weekly
Lixisenatide	Exendin-4–based, C-terminal polylysine chain	Used exclusively as part of a fixed-dose combination with insulin glargine (iGlarLixi^®^)
Liraglutide (Victoza^®^)	GLP-1–based, fatty acid modification at position 26, albumin binding	Once daily
Dulaglutide (Trulicity^®^)	GLP-1–based, G at position 8, 2 copies + IgG4 Fc-fragment	Once weekly
Albiglutide (Eperzan^®^)	GLP-1–based, G at position 8, 2 copies + albumin	Once weekly (withdrawn from the market)
Semaglutide (Ozempic^®^)	GLP-1–based, Aib at position 8, fatty acid modification at position 26, albumin binding	Once weekly
Semaglutide (oral) (Rybelsus^®^)	GLP-1–based, Aib at position 8, fatty acid modification at position 26, albumin binding, SNAC for absorption	Once daily
Tirzepatide (Mounjaro^®^)	Dual GIP/GLP-1 agonist, Aib at position 2, fatty acid modification at position 20	Once weekly

**Table 2:** Clinical trials evaluating GLP-1RAs and dual/triple incretin agonists in cardio–renal–metabolic disorders.

Clinical Trial	Medication	Mechanism	Disease	Phase
NCT01720446—SUSTAIN-6	Semaglutide	GLP-1RA	MACE, CKD	Phase III
NCT02692716—PIONEER-6	Semaglutide	GLP-1RA	MACE, CKD	Phase III
NCT03574597—SELECT	Semaglutide	GLP-1RA	MACE	Phase III
NCT03914326—SOUL	Semaglutide	GLP-1RA	MACE, CKD	Phase III
NCT03819153—FLOW	Semaglutide	GLP-1RA	CKD	Phase III
NCT04788511—STEP-HFpEF	Semaglutide	GLP-1RA	CVD, HFpEF	Phase III
NCT04916470—STEP-HFpEF DM	Semaglutide	GLP-1RA	CVD, HFpEF	Phase III
NCT04822181—ESSENCE	Semaglutide	GLP-1RA	MASH, NASH	Phase III
NCT02970942—SEMA-NASH	Semaglutide	GLP-1RA	MASH, NASH	Phase II
NCT01179048—LEADER	Liraglutide	GLP-1RA	MACE	Phase III
NCT01237119—LEAN	Liraglutide	GLP-1RA	MASH, NASH	Phase II
NCT01394952—REWIND	Dulaglutide	GLP-1RA	MACE	Phase III
NCT01621178—AWARD-7	Dulaglutide	GLP-1RA	CKD	Phase III
NCT02465515—Harmony Outcomes	Albiglutide	GLP-1RA	MACE	Phase IV
NCT01144338—EXSCEL	Exenatide	GLP-1RA	MACE	Phase III
NCT01147250—ELIXA	Lixisenatide	GLP-1RA	MACE	Phase III
NCT05869903—ATTAIN-1	Orforglipron	GLP-1RA	CVD	Phase III
NCT03730662—SURPASS-4	Tirzepatide	GLP-1/GIP RA	MACE, ASCVD	Phase III
NCT04255433—SURPASS-CVOT	Tirzepatide	GLP-1/GIP RA	MACE, CVD	Phase III
NCT04184622—SURMOUNT-1	Tirzepatide	GLP-1/GIP RA	MACE, HFpEF, CVD MASH, CKD	Phase III Phase II
NCT04847557—SUMMIT	Tirzepatide	GLP-1/GIP RA	CVD, HFpEF	Phase III
NCT04166773—SYNERGY-NASH	Tirzepatide	GLP-1/GIP RA	MASH, NASH	Phase II
NCT05203237—VK2735 Study	VK2735	GLP-1/GIP RA	MASH, NASH	Phase I
NCT04667377—Survodutide (BI 456906) Study	Survodutide	GLP-1/glucagon RA	MASH	Phase II
NCT06077864—SYNCHRONIZE-CVOT	Survodutide	GLP-1/glucagon RA	CVD, CKD	Phase III
NCT04771273—Survodutide (BI 456906) NASH and Fibrosis Study	Survodutide	GLP-1/glucagon RA	MASH, NASH	Phase II
NCT05295875—MOMENTUM	Pemvidutide	GLP-1/glucagon RA	MASH, MASLD	Phase II
NCT03496298—AMPLITUDE-O	Efpeglenatide	GLP-1/glucagon RA	MACE, CKD	Phase III
NCT03486392—JNJ-64565111 Study	Efinopegdutide	GLP-1/glucagon RA	MASH, MASLD	Phase II
NCT05607680—GLORY-1	Mazdutide	GLP-1/glucagon RA	CKD	Phase I
NCT05929066—TRIUMPH-1	Retatrutide	GLP-1/GIP/glucagon RA	CKD	Phase II
NCT06383390—TRIUMPH-Outcomes	Retatrutide	GLP-1/GIP/glucagon RA	CVD, CKD	Phase III
NCT03374241—HM15211 Study	Efocipegtrutide	GLP-1/GIP/glucagon RA	MASH	Phase II

## Data Availability

No new data were created or analyzed in this study.

## Abbreviations

The following abbreviations are used in this manuscript:

AC	Adenylate Cyclase
ACE	Angiotensin-Converting Enzyme
ADA	American Diabetes Association
Akt	Protein Kinase B
ALT	Alanine Aminotransferase
ASCVD	Atherosclerotic Cardiovascular Disease
AST	Aspartate Aminotransferase
cAMP	Cyclic Adenosine Monophosphate
CNS	Central Nervous System
COPD	Chronic Obstructive Pulmonary Disease
CRMS	Cardio–Renal–Metabolic Syndrome
CVOT	Cardiovascular Outcome Trial
DKD	Diabetic Kidney Disease
DN	Diabetic Nephropathy
DPP-4	Dipeptidyl Peptidase-4
EPAC	Exchange Protein Directly Activated By Camp
FDA	Food And Drug Administration
GCGR	Glucagon Receptor
GFR	Glomerular Filtration Rate
GIP	Glucose-Dependent Insulinotropic Polypeptide
GIPR	GIP Receptor
GLP-1	Glucagon-Like Peptide-1
GLP-1R	GLP-1 Receptor
GLP-1RA	GLP-1 Receptor Agonist
GLUT-4	Glucose Transporter Type 4
GPCR	G Protein-Coupled Receptor
HbA1c	Glycated Hemoglobin
HDL	High-Density Lipoprotein
HFpEF	Heart Failure With Preserved Ejection Fraction
HSA	Human Serum Albumin
IR	Insulin Resistance
IRS-1	Insulin Receptor Substrate 1
LDL	Low-Density Lipoprotein
MACE	Major Adverse Cardiovascular Events
MASH	Metabolic Dysfunction-Associated Steatohepatitis
MASLD	Dysfunction-Associated Steatotic Liver Disease
MI	Myocardial Infarction
NAFLD	Non-Alcoholic Fatty Liver Disease
NAION	Non-Arteritic Anterior Ischemic Optic Neuropathy
PEG	Polyethylene Glycol
PI3K	Phosphoinositide 3-Kinase
PLGA	Poly(Lactic-Co-Glycolic Acid)
PKA	Protein Kinase A
PKCβ	Protein Kinase C Beta
PPAR-α	Peroxisome Proliferator-Activated Receptor Alpha
RAAS	Renin–Angiotensin–Aldosterone System
Ro5	Lipinski’s Rule Of Five
SGLT2	Sodium-Glucose Cotransporter 2
SGLT2i	Sodium–Glucose Cotransporter 2 Inhibitors
SNAC	Sodium N-[8-(2-Hydroxybenzoyl)Aminocaprylate]
SPPS	Solid-Phase Peptide Synthesis
T2DM	Type 2 Diabetes Mellitus
WHO	World Health Organization
(AEEA)_2_	[2-(2-(2-aminoethoxy) ethoxy) acetic acid]_2_
(Oda)_2_	(2,2′-Oxydiacetic acid)_2_
